# Soil texture as a key driver of polycyclic aromatic hydrocarbons (PAHs) distribution in forest topsoils

**DOI:** 10.1038/s41598-021-94299-x

**Published:** 2021-07-19

**Authors:** Stanisław Łyszczarz, Jarosław Lasota, Maria Magdalena Szuszkiewicz, Ewa Błońska

**Affiliations:** 1 Department of Ecology and Silviculture, Faculty of Forestry, University of Agriculture in Krakow, 29 Listopada 46, 31-425 Kraków, Poland; 2 Institute of Environmental Engineering, Polish Academy of Sciences, 34 M. Skłodowskiej-Curie St., 41-819 Zabrze, Poland

**Keywords:** Environmental chemistry, Environmental impact, Ecosystem ecology, Forest ecology, Forestry, Microbial ecology

## Abstract

Due to the dynamic development of civilization and the increasing demand for energy, pollution by harmful chemicals, including polycyclic aromatic hydrocarbons (PAHs) compounds, is a serious threat to forest soils. The aim of the study was to determine the role of texture in the distribution of polycyclic aromatic hydrocarbons (PAHs) and trace elements in forest soils. The areas with different texture ranging from sand through sandy loam to silt loam were selected for the study. The study was carried out in the Chrzanów Forest District in southern Poland (50° 7′ 18 N; 19° 31′ 29 E), which in one of the most intensive industrial emission zones in Europe. The soil samples for properties determination were collected from locations distributed on a regular grid 100 × 100 m (20 points). The samples were collected from the humus horizon (0–10 cm) after removing organic horizon. Basic chemical properties, heavy metal content, polycyclic aromatic hydrocarbons (PAHs) content and magnetic susceptibility values were determined in soil samples. Additionally, enzymatic activity and microbiological biomass was determined in the samples. Our study confirmed the importance of texture in PAHs distribution. A strong correlation between PAHs content and silt content in the soils studied was noted. The regression tree analysis confirmed the importance of the silt content, followed by soil organic carbon in PAHs distribution. Organic carbon content and nitrogen content played a predominant role in controlling the microbial activity. In our study, we did not note a relationship between enzymatic activity, microbiological soil biomass and the amount of PAHs. This may be due to the effective sorption and immobilization of PAHs by particles of fine fractions, especially silt. Obtained results confirmed the usefulness of magnetic susceptibility in the assessment of heavy metals contamination of forest soils. We noted high correlation between magnetic susceptibility value and heavy metals content. Moreover, the relationship between magnetic susceptibility and soil texture of the topsoil was also observed.

## Introduction

Polycyclic aromatic hydrocarbons (PAHs) are characterized by high toxicity and mutagenicity^1^. As the number of benzene rings and molecular weight increase, their water solubility and biodegradability decrease, making them more toxic^2^. Polycyclic aromatic hydrocarbons in the soil environment are characterized by high durability, low mobility and high bioaccumulation capacity^3^. PAH compounds are classified as persistent organic pollutants^4^ and the US Environmental Protection Agency has identified PAHs as one of the main ecosystem pollution problems, recommending monitoring of their content in plants, soil and aquatic environments^5^. Currently, as a result of increased human activity, anthropogenic PAHs emissions have increased significantly^6,7^. PAHs are formed mainly as a result of technological use of fossil fuels in high temperature conditions. The factors determining the accumulation of PAHs in soils are: distance from the emission source, climatic conditions, soil organic matter^8–10^. According to Terytze et al.^11^, PAHs are characterized by a strong sorption affinity to soil organic matter. Extensive studies have been done with regard to the relationship of PAHs with total organic carbon (TOC) and elemental carbon (EC) in different environmental matrices^12^. According to Duan et al.^13^, an important role in PAHs accumulation in the soil environment is played by meso- and macrophores and clay colloids responsible for sorption of pollutants. Clay can have a significant impact on PAHs sequestration in soil^14^. The addition of exogenous-rich carbon material such as biochar to the soil significantly changes the behavior and sorption potential of PAHs in the soil^15^. The type of stand significantly affects the content of polycyclic aromatic hydrocarbons in forest soils^16^. Lasota and Błońska^17^ experiment provided evidence that the quantity and quality of soil organic matter plays an important role in controlling PAH amounts.

Monitoring by means of the characteristics of microbiological and biochemical properties of soils is successfully used in assessing the degree of soil contamination^18,19^. Enzyme activity could be a good indicator of soil quality because it is sensitive and reflects biological situation in the soil and is strongly correlated with important soil characteristics, such as organic matter and soil texture^20^. Weaker degradation of hydrocarbons is associated with lower biological activity of soils. Lasota and Błońska^17^ noted in the mull humus type lower content of PAHs and at the same time the highest biological activity confirmed by high dehydrogenase activity. PAH degradation may not be directly correlated by the soil enzyme activity but related to soil indigenous population of PAH-degrading microorganism^21^.

Soil magnetic susceptibility (MS) is an important parameter in pollution studies owing to its relationship with atmospheric deposition^22^. This works well due to the concomitance of technogenic magnetic particles (TMPs) with heavy metals^23^. It is well-known that the main sources of TMPs and heavy metals are high-temperature technological and combustion processes^24^. The deposition and accumulation of TMPs into topsoil leads to enhancement of magnetic signal and elevation of heavy metal contents^25–27^. The presence of TMPs in soil can be easily detected via magnetic susceptibility measurements (especially in the highly urbanized and industrialized areas). Moreover, magnetic parameters can be used to study various processes, which take place in soils and where magnetic minerals maybe used as indicators of changes and transformation^28–32^. Few of the studies show the state of contamination with heavy metals and PAHs in forest soils in connection with the magnetic susceptibility parameters^33^.

The aim of the study is to determine the role of texture in the distribution of polycyclic aromatic hydrocarbons (PAHs) in forest soils. The areas with different texture ranging from sand through sandy loam to silt loam were selected for the study. The following research hypotheses were tested: (1) the content of silt strongly determined the distribution of polycyclic aromatic hydrocarbons in forest soils; (2) organic carbon plays an important role in the accumulation of PAHs and formation of the enzymatic activity of forest soils; (3) magnetic susceptibility is a useful tool in the assessment of contamination of forest topsoil with different texture.

## Material and methods

### Study area and soil sampling

**Figure 1:**
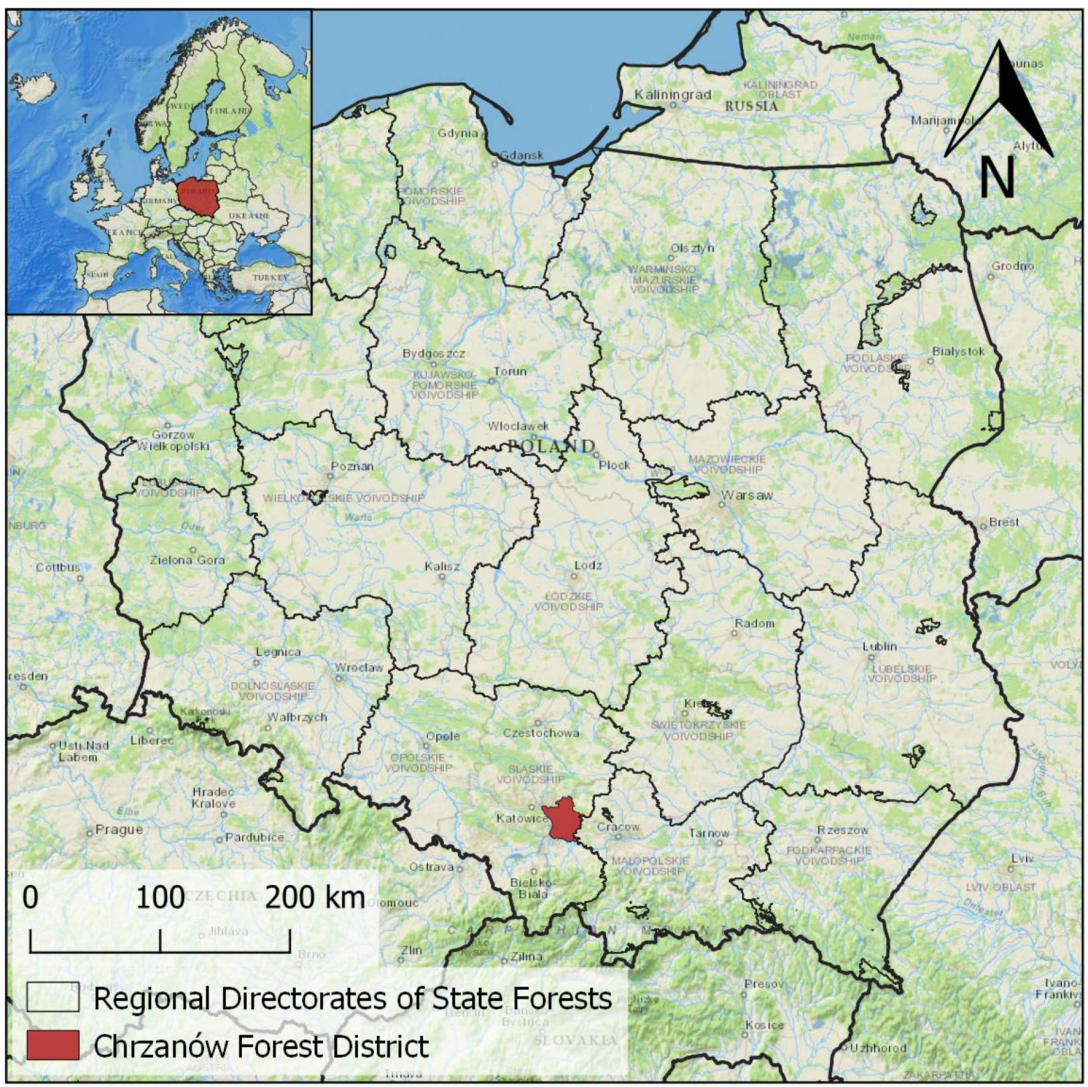
Map of the study location**.** QGIS 3.16 software (https://www.esri.com/en-us/about/about-esri/overview) was used to create the map. The layer of the location map was the World Topographic Map basemap within QGIS 3.16 software. The map is credited to: Esri, HERE, DeLorme, Intermap, increment P Corp., GEBCO, USGS, FAO, NPS, NRCAN, GeoBase, IGN, Kadaster NL, Ordnance Survey, Esri Japan, METI, Esri China (Hong Kong), swisstopo, MapmyIndia, OpenStreetMap contributors, and the GIS User Community.

The research was carried out in the Chrzanów Forest District in southern Poland (50° 7′ 18 N; 19° 31′ 29 E) (Fig. 1). The study plots with different texture were selected for the analysis (from sand by sandy loam to silt loam). The research area was dominated by a pine-oak stand of a similar age, i.e. 60–80 years. The average annual temperature for this area was 7.8 °C, and the average annual rainfall is 658 mm. The study area was located in an industrial emission zone, which comes mainly from Upper Silesian Industrial District. The elevated content of polycyclic aromatic hydrocarbons (PAH) in the studied soils was confirmed by earlier research^16^. The selection of the study plots was made during field observation. Before selecting the research plot, the authors, using a soil auger, checked the variability of the soil cover and assessed the accompanying vegetation. The study area was dominated by Stagnosols^34^, created on water and glacial formations.

According to the Report on the state of the environment in the Małopolska Voivodeship in the period 2012–2017, the average annual benzo(a)pyrene (BaP) concentrations exceeded acceptable standards (1 ng m^3^) across the whole Chrzanów Forest District. The average annual concentration of BaP near air pollution station in the neighborhood of the research area was 5.5 ng m^3^. The average annual concentrations of particulate matter < 10 µm in diameter (PM10) over the entire range of the Chrzanów Forest District was 34 µg m^3^. The mean annual concentrations of particulate matter < 2.5 µm in diameter (PM2.5) in the atmosphere exceeded the admissible standard (according to health protection criteria) of 27 µg m^3^ across the whole of the Chrzanów Forest District.

**Figure 2:**
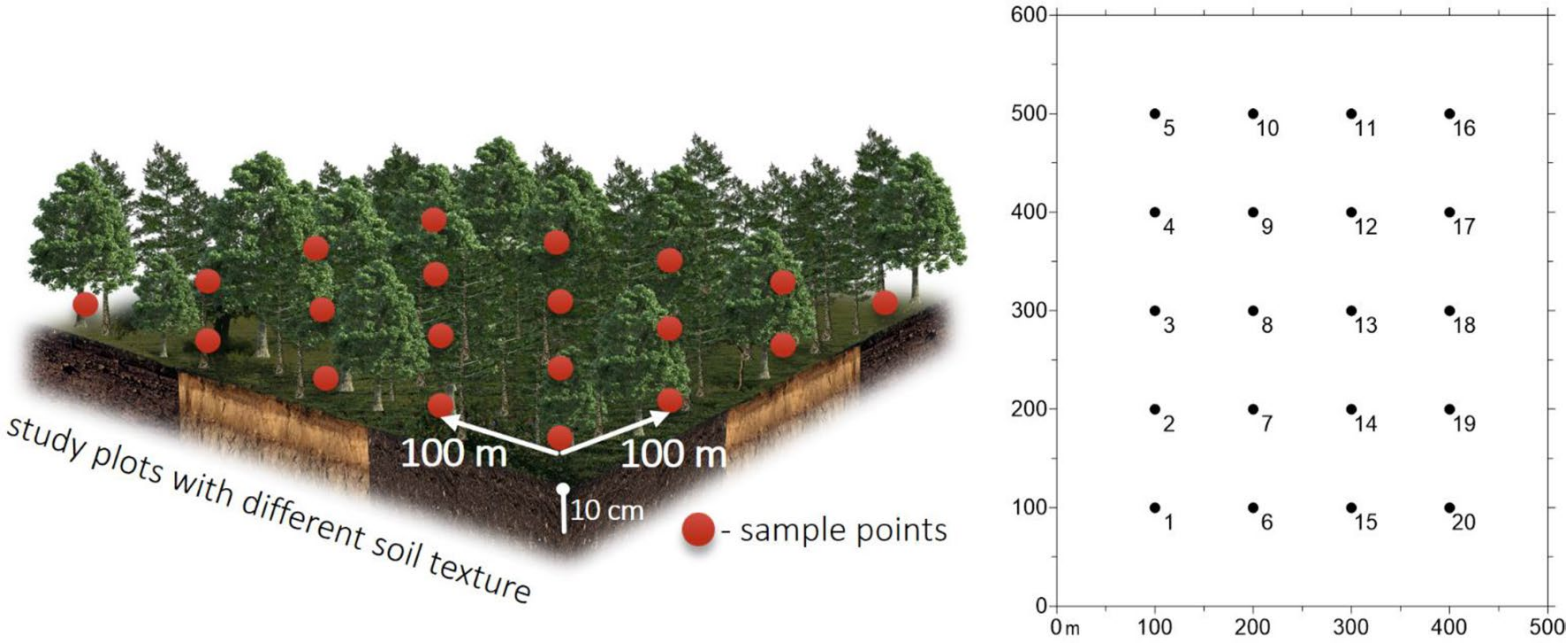
Scheme of the sampling points locations. GIMP 2.10.20 (https://www.gimp.org), Surfer 10 software and elements from the Freepik website (https://www.freepik.com) were used to create the structure of the figure.

Soil samples for laboratory analysis were collected in August 2018. The soil samples for properties determination were collected from locations distributed on a regular grid 100 × 100 m (20 points) (Fig. 2). The samples were collected from the humus mineral horizon (10 cm deep) after removing organic horizon. In all the cases, the samples for the study were collected from 4 sub-stands of soil. For determination of enzymes activity, microbial biomass and PAHs content, fresh samples of natural moisture were sieved through a sieve (ø 2 mm) and stored at 4 °C in the dark before analysis. The results were analyzed in three groups of surfaces separated on the basis of texture (I group with sand texture, II group with sandy loam texture and III group with silt loam).

### Laboratory analysis

Freshly collected soil samples were dried and then sieved through a 2 mm mesh sieve. The particle size distribution was analyzed using a laser diffraction method (Analysette 22, Fritsch, Idar-Oberstein, Germany). The pH of soil samples in H_2_O and KCl was determined by potentiometric method. An elemental analyzer (LECO CNS TrueMac Analyzer (Leco, St. Joseph, MI, USA)) was used to determine carbon (C) and nitrogen (N). By ICP method (ICP-OES Thermo iCAP 6500 DUO, Thermo Fisher Scientific, Cambridge, U.K.), the concentration of basic cations and the contents of Cd, Cr, Cu, Ni, Pb, Zn were determined. The contents of the studied heavy metals were determined after digestion in a 2:1 solution of concentrated nitric acid and perchloric acid.

From each soil sample collected, 10 g of soil was taken after mixing. Polycyclic aromatic hydrocarbons were extracted from the tested amount of soil in 70 ml of 2-propanol. The samples were centrifuged (4500, 5 min) and the supernatant was collected and later subjected to solid phase extraction (5 ml/min)—Solid Phase Extraction (Chromabond Cn/SiOH). The resulting extraction residue was dissolved in acetonitrile and analyzed by HPLC, equipped with a Dionex UltiMate 3000 Column Compartment—C18 5 μm, 4.6 × 100 mm HPLC column and a fluorescence detector (FLD). Water (A) and acetonitrile (B) was the mobile phase with a flow rate of 1 ml/min. A PAH mixture standard (CRM 47940) with a concentration of 10 μg/ml was used to calibrate the analyses. Calibration solutions had concentrations of 0.1 μg/ml, 0.2 μg/ml, 0.5 μg/ml, 1 μg/ml, and 2 μg/ml. Samples of the prepared solutions were dosed onto the chromatograph column. The generated chromatograms determined the standard curve. Later, soil samples were dosed in triplicate. At the end of each analysis, an "unknown" sample was applied, which was a 0.1 μg/ml calibration solution used as control material. Thirteen polycyclic aromatic hydrocarbons were analyzed in the soil samples: naphthalene (Nft), fluorene (Flu), phenanthrene (Phe), anthracene (Ant), fluoranthene (Flt), pyrene (Pyr), benzo(a)anthracene (BaA), and chrysene (Chr), benzo(k)fluoranthene (BkF), benzo(b)fluoranthene (BbF), benzo(a)pyrene (BaP), indeno(1,2,3-c,d)pyrene (IcdP), and bezo(g,h,i)perylene (BghiP).

Enzymatic activity was determined using fluorogenic labeled substrates^35,36^. The fluorogenic enzyme substrates that were used for analysis were based on 4-methylumbelliferone (MUB). The following substrates were used: MUB-β-d-cellobioside for β-d-cellobiosidase (CB), MUB-β-d-xylopyranoside for xylanase (XYL), MUB-N-acetyl-β-d-glucosaminide for N-acetyl-β-d-glucosaminidase (NAG), MUB-β-d-glucopyranoside for β-glucosidase (BG)^37^. 2.75 g of soil from each sample was measured and mixed with 92 ml of universal buffer (pH 6.0). The resulting soil solution was pipetted into wells located on a microscope plate that contained the substrate and a modified universal buffer. Fluorescence was determined by incubating the soil suspension determined for 1.5 h at 35 °C in 96-well microplates (Puregrade, Germany). Fluorescence was then immediately determined on a multi-detector plate reader (SpetroMax), with excitation at 355 nm and emission at 460 nm. Carbon in microbial biomass (MBC) and nitrogen in microbial biomass (MBN) were determined by fumigation and extraction^38,39^.

By measuring magnetic susceptibility in the studied soil samples, the proportion of magnetic particles was determined. In the laboratory, low-field magnetic susceptibility (volumetric—κ) was measured at two different frequencies: low (465 Hz) and high (4650 Hz) using a Bartington MS2 magnetic susceptibility meter equipped with an MS2B sensor (Bartington Instruments Ltd.) Based on the measurements of κ values, the bulk magnetic susceptibility (χ) and the percentage frequency-dependent magnetic susceptibility (χ_fd_) were determined^40^.

### Statistical analysis

ANOVA test was used to evaluate the differences between the mean values of the soil properties. Pearson correlation coefficients for the soil characteristics were calculated. The principal component analysis (PCA) method was used to evaluate the relationships between soil properties and PAH content. The classification and regression tree (C&RT) approach was applied to estimation of soil properties influence on PAH content. Differences with P < 0.05 were considered statistically significant. All analyses were performed using Statistica 12 software.

## Results

### Physicochemical properties

The soils studied were divided into three groups, which differed significantly in sand, silt and clay content. The highest silt content was recorded in soils with sandy loam and silt loam texture (Table 1). No statistically significant differences in pH of the soils studied were noted. Average pH in H_2_O of soils with sand, sandy loam and silt loam texture was 4.01, 4.21 and 4.06 (Table 1). Soils with the texture of sandy loam and silt loam were characterized by statistically significantly higher carbon and nitrogen content compared to soils with sand texture (Table 1). The highest carbon content was recorded in soils with sandy loam grain size (6.43%) and the lowest in soils with sand grain size (2.95%). The mean nitrogen content in soils with sandy loam and silt loam texture is 0.34% and in sandy soils it is significantly lower at a level of 0.12%. No statistically significant differences in C/N of the soils studied were recorded (Table 1). The content of alkaline cations was significantly lower in sandy soils (Table 1). In soils with different grain sizes, different enzymatic and microbial biomass of C and N was noted (Table 2).

**Table 1:** Basic properties of analysed soils.

Class	Sand	Silt	Clay	pH H_2_O	pH KCl	C	N	C/N	Ca	K	Mg	Na
Sand	93 ± 2^b^	6 ± 2^b^	1 ± 1^b^	4.01 ± 0.21^a^	3.17 ± 0.25^a^	2.95 ± 1.47^b^	0.12 ± 0.05^b^	25.8 ± 5.6^a^	0.27 ± 0.22^b^	0.05 ± 0.02^b^	0.07 ± 0.04^b^	0.01 ± 0.01^a^
Sandy loam	57 ± 10^a^	37 ± 9^a^	5 ± 2^a^	4.21 ± 0.50^a^	3.50 ± 0.43^a^	6.43 ± 2.35^a^	0.34 ± 0.14^a^	19.8 ± 5.9^a^	1.75 ± 0.77^a^	0.15 ± 0.07^a^	0.35 ± 0.22^a^	0.02 ± 0.01^a^
Silt loam	41 ± 2^a^	53 ± 2^a^	4 ± 1^a^	4.06 ± 0.23^a^	3.43 ± 0.29^a^	5.86 ± 0.39^a^	0.34 ± 0.09^a^	18.5 ± 5.9^a^	0.89 ± 0.47^ab^	0.12 ± 0.03^a^	0.25 ± 0.11^ab^	0.02 ± 0.01^a^

Mean ± standard deviation; sand, silt and clay content (%); carbon and nitrogen content (%); Ca, K, Mg and Na content (cmol(+)/kg).

Small letters in the upper index of the mean values mean significant differences between texture class.

**Table 2:** Enzyme activities of soil and microbial biomass carbon and nitrogen.

Class	CB	BG	NAG	XYL	MBC	MBN
Sand	9.95 ± 3.46^a^	9.15 ± 8.15^a^	9.56 ± 6.81^a^	11.69 ± 8.76^a^	348.71 ± 186.94^a^	39.30 ± 30.71^b^
Sandy loam	16.62 ± 14.36^a^	21.95 ± 13.18^a^	16.93 ± 8.62^a^	15.29 ± 10.76^a^	571.20 ± 285.82^a^	63.49 ± 36.89^ab^
Silt loam	14.74 ± 6.91^a^	19.44 ± 11.30^a^	20.37 ± 10.20^a^	14.57 ± 10.54^a^	446.50 ± 54.13^a^	95.02 ± 28.29^a^

Mean ± standard deviation.

Small letters in the upper index of the mean values mean significant differences between texture class.

*CB* β-d-cellobiosidase, *BG* β-glucosidase, *NAG* N-acetyl-β-d-glucosaminidase, *XYL* xylanase (nmol MUB/gd s/h), *MBC* microbial biomass carbon, *MBN* microbial biomass nitrogen (µg/g).

### Biochemical properties

High activity of CB, BG, NAG and XYL was recorded in soils with sandy loam and silt loam texture (Table 2). The differences in enzymatic activity were not statistically significant. In the case of microbial biomass C and N, the results were similar to the enzymatic activity. High microbial biomass C and N was recorded in soils with a finer grain size. The microbial biomass N was statistically significantly higher in soils with silt loam texture (Table 2). Enzymatic activity and microbial biomass C and N correlated significantly with C, N and Na content (Table 3). In case of CB, BG, MBC and MBN a positive correlation with potassium content was noted. No significant correlations of biochemical parameters with heavy metals, magnetometry and PAHs content were found (Table 3).

**Table 3:** Correlation between biochemical properties and basic properties of soils.

	CB	BG	NAG	XYL	MBC	MBN
N	0.6234*	0.5516*	0.5321*	0.4851*	0.6076*	0.6392
C	0.6083*	0.4818*	0.3877	0.4936*	0.6272*	0.5783
Sand	− 0.3690	− 0.4212	− 0.3241	− 0.2795	− 0.4041	− 0.3635
Silt	0.3903	0.4484*	0.3541	0.3002	0.4004	0.3678
Clay	0.1094	0.1015	− 0.0183	0.0366	0.3451	0.2460
pH in H_2_O	− 0.2914	− 0.1549	− 0.2329	− 0.4048	− 0.0870	0.0161
pH in KCl	− 0.0528	0.1370	0.0645	− 0.1986	0.1879	0.2857
Ca	− 0.1498	− 0.1244	− 0.1456	− 0.3139	− 0.0962	0.0173
K	0.5456*	0.5059*	0.4410	0.4351	0.5995*	0.6072*
Mg	0.0062	0.0142	− 0.0020	− 0.1739	0.0442	0.1610
Na	0.7006*	0.7628*	0.6017*	0.5191*	0.6510*	0.6109*
PAHs	0.0114	0.0465	− 0.0883	− 0.0678	0.1144	− 0.0087
Cd	0.2406	0.1309	0.0607	0.0688	0.0729	0.1067
Co	0.4145	0.4498*	0.4387	0.2139	0.2599	0.2966
Cr	0.4060	0.3896	0.3929	0.2261	0.3049	0.3565
Cu	0.3650	0.3432	0.3605	0.2121	0.3154	0.3385
Mn	0.0820	0.1250	− 0.0060	− 0.1687	0.0608	− 0.0236
Ni	0.1007	0.2463	0.2543	0.0656	− 0.0418	− 0.1686
Pb	0.5166*	0.4369	0.4099	0.3470	0.4459*	0.4393
Zn	0.3731	0.3884	0.3136	0.1219	0.2505	0.2820

*p < 0.05.

### Polycyclic aromatic hydrocarbons and heavy metals content

**Figure 3:**
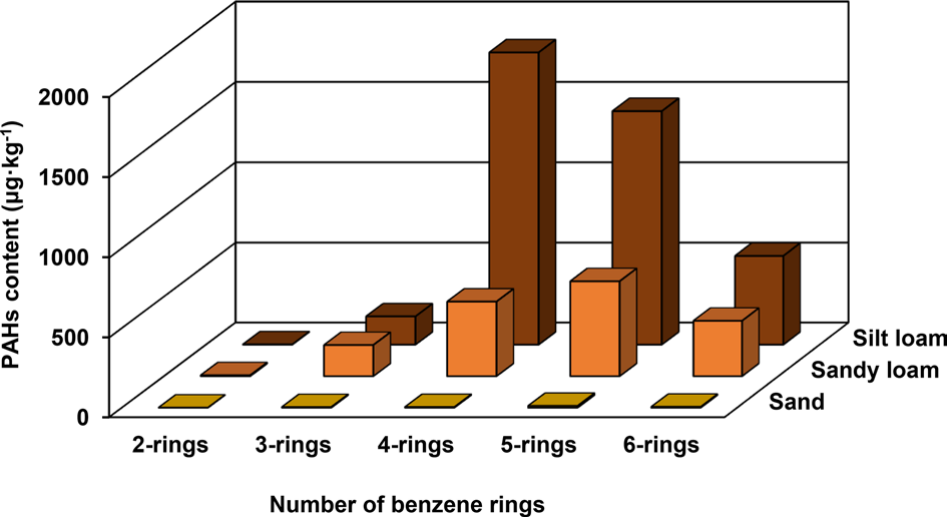
PAHs content (μg/kg) in soil depending on the benzene rings, was generated using Statistica 13 software.

**Figure 4:**
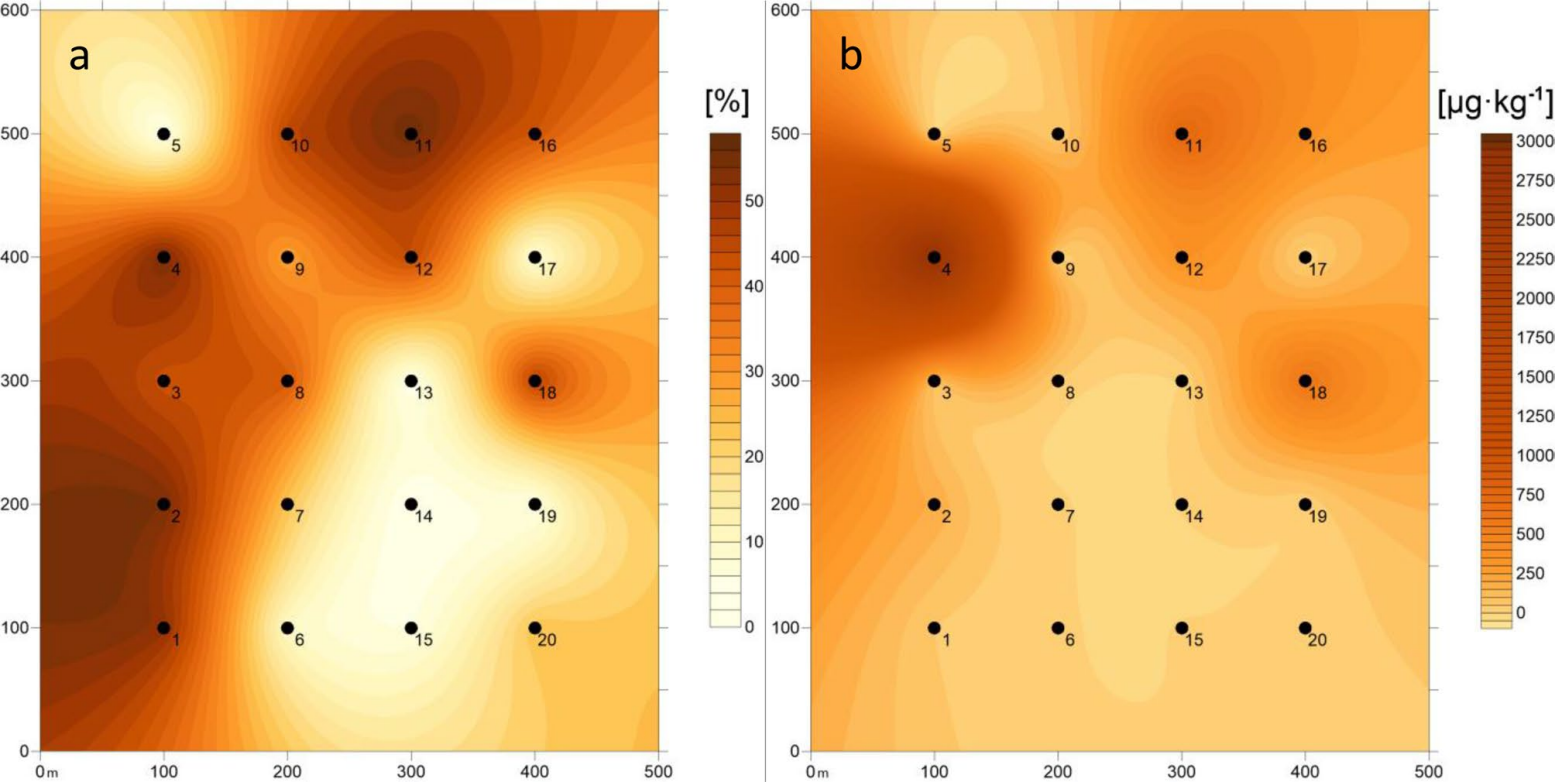
Spatial distribution of silt (**a**) and PAHs (**b**) content in soils, was plotted using Surfer 10 software.

The highest content of heavy metals was recorded in the soils with sandy loam and silt loam texture (Table 4). The differences were statistically significant. The relationship between magnetic susceptibility and soil texture of the topsoil was also observed. Magnetic susceptibility was directly correlated with the percentage of silt and inversely correlated with the percentage of sand, suggesting that magnetic particles are associated with finer soil fraction. Magnetic susceptibility gradually decreases with sand fraction content (Table 4), i.e. the highest mean values of χ were recorded in soils with silt loam texture (59.82 × 10^–8^ m^3^/kg), lower in soils with sandy loam texture (53.14 × 10^–8^ m^3^/kg) and the lowest in sandy soils (16.44 × 10^–8^ m^3^/kg). In contrast, the percentage values of frequency-dependent magnetic susceptibility (χ_fd_) were comparable, regardless of soil texture and show similar patterns for study soil fraction (i.e. did not exceed 2%, Table 4). The highest sum of PAHs was recorded in soils with heavy texture (sandy loam and silt loam). The mean sum of PAHs was 3.62 μg/kg in soils with sand texture, 178.35 μg/kg in sandy loam and 1369.41 μg/kg in silt loam texture. These values were significantly higher compared to sandy soils (Table 4). In soils with a higher silt content, high contents of 4, 5 and 6 ring hydrocarbons were recorded (Fig. 3). In the case of most heavy metals, magnetic susceptibility and PAHs, a significant positive correlation with silt content and a negative correlation with sand content was observed (Table 5, Fig. 4). For Cr, Cu, Pb and Zn, a positive correlation with clay content was noted (Table 5). Heavy metals content and magnetic susceptibility were strongly positively correlated with N, C and selected alkaline cations (K, Mg and Na) (Table 5). Moreover, the high and significant correlation between χ and Cd, Co, Cr, Cu, Mn, Pb and Zn was stated (Table 5).

**Table 4:** Heavy metals content, magnetic susceptibility and PAHs content in soils.

Class	Cd	Co	Cr	Cu	Mn	Ni	Pb	Zn	χ	χ_fd_	ƩPAHs
Sand	0.67 ± 0.60^b^	0.33 ± 0.15^b^	4.86 ± 2.06^b^	18.13 ± 7.45^b^	18.25 ± 9.30^b^	6.73 ± 2.96^b^	53.16 ± 27.96^b^	22.69 ± 14.44^b^	16.44 ± 7.37^b^	1,47 ± 0.12^b^	3.62 ± 2.37^b^
Sandy loam	1.55 ± 0.65^a^	1.92 ± 1.22^a^	22.81 ± 13.82^a^	34.07 ± 14.83^a^	97.89 ± 61.45^a^	6.96 ± 2.25^a^	197.83 ± 127.96^a^	83.64 ± 40.14^a^	53.14 ± 40.14^a^	1,74 ± 1.10^a^	178.35 ± 115.11^a^
Silt loam	1.35 ± 0.25^a^	2.93 ± 1.88^a^	22.14 ± 11.68^a^	35.91 ± 5.56^a^	181.69 ± 119.86^a^	7.81 ± 1.37^a^	162.38 ± 37.71^a^	84.06 ± 40.62^a^	59.82 ± 32.50^a^	1.19 ± 0.50^a^	1369.41 ± 1003.39^a^

Mean ± standard deviation; χ—mass magnetic susceptibility (10^–8^ m^3^/kg); χ_fd_ (%)—frequency-dependent magnetic susceptibility; Cd, Co, Cr, Cu, Mn, Ni, Pb and Zn (mg/kg); ƩPAHs sum of polycyclic aromatic hydrocarbons content (μg/kg).

**Table 5:** Correlation between heavy metals content, PAHs content, magnetic susceptibility and soil properties.

	Cd	Co	Cr	Cu	Mn	Ni	Pb	Zn	χ	PAHs
N	0.5625*	0.7640*	0.8775*	0.7434*	0.4303	0.1207	0.8754*	0.7981*	0.7341*	0.0971
C	0.6605*	0.5139*	0.6531*	0.6445*	0.3019	0.1306	0.8110*	0.6271*	0.6042*	0.2561
C/N	− 0.1513	− 0.7007*	− 0.7085*	− 0.5109*	− 0.4170	− 0.0055	− 0.4440*	− 0.6330*	− 0.5307*	0.1319
Sand	− 0.5205*	− 0.7026*	− 0.6662*	− 0.6187*	− 0.5031*	− 0.1723	− 0.6365*	− 0.6801*	− 0.5083*	− 0.4640*
Silt	0.5209*	0.7186*	0.6672*	0.6079*	0.5026*	0.1840	0.6246*	0.6845*	0.5223*	0.4696*
Clay	0.4169	0.4042	0.5055*	0.5721*	0.3948	− 0.0002	0.5983*	0.4971*	0.2799	0.3184
pH in H_2_O	0.0208	0.1931	0.1992	− 0.1385	0.2226	− 0.3404	− 0.0789	0.2724	0.0655	− 0.1653
pH in KCl	0.0616	0.4228	0.3952	0.0537	0.2933	− 0.2504	0.0813	0.4303	0.3204	− 0.1450
Ca	0.2674	0.3149	0.4196	0.0998	0.3368	− 0.2073	0.2541	0.4576*	0.1363	− 0.1177
K	0.5226*	0.6680*	0.8664*	0.7150*	0.3428	0.0961	0.8879*	0.7639*	0.6482*	0.0718
Mg	0.4736*	0.5591*	0.6784*	0.3713	0.4916*	− 0.1401	0.5051*	0.6991*	0.4159	− 0.1187
Na	0.5581*	0.7437*	0.7153*	0.6110*	0.4702*	0.1887	0.6809*	0.7783*	0.6976*	0.2519
χ	0.5508*	0.7539*	0.7450*	0.7262*	0.5296*	0.2148	0.6814*	0.7622*	1.000	− 0.0347

*p < 0.05.

### Correlations

**Figure 5:**
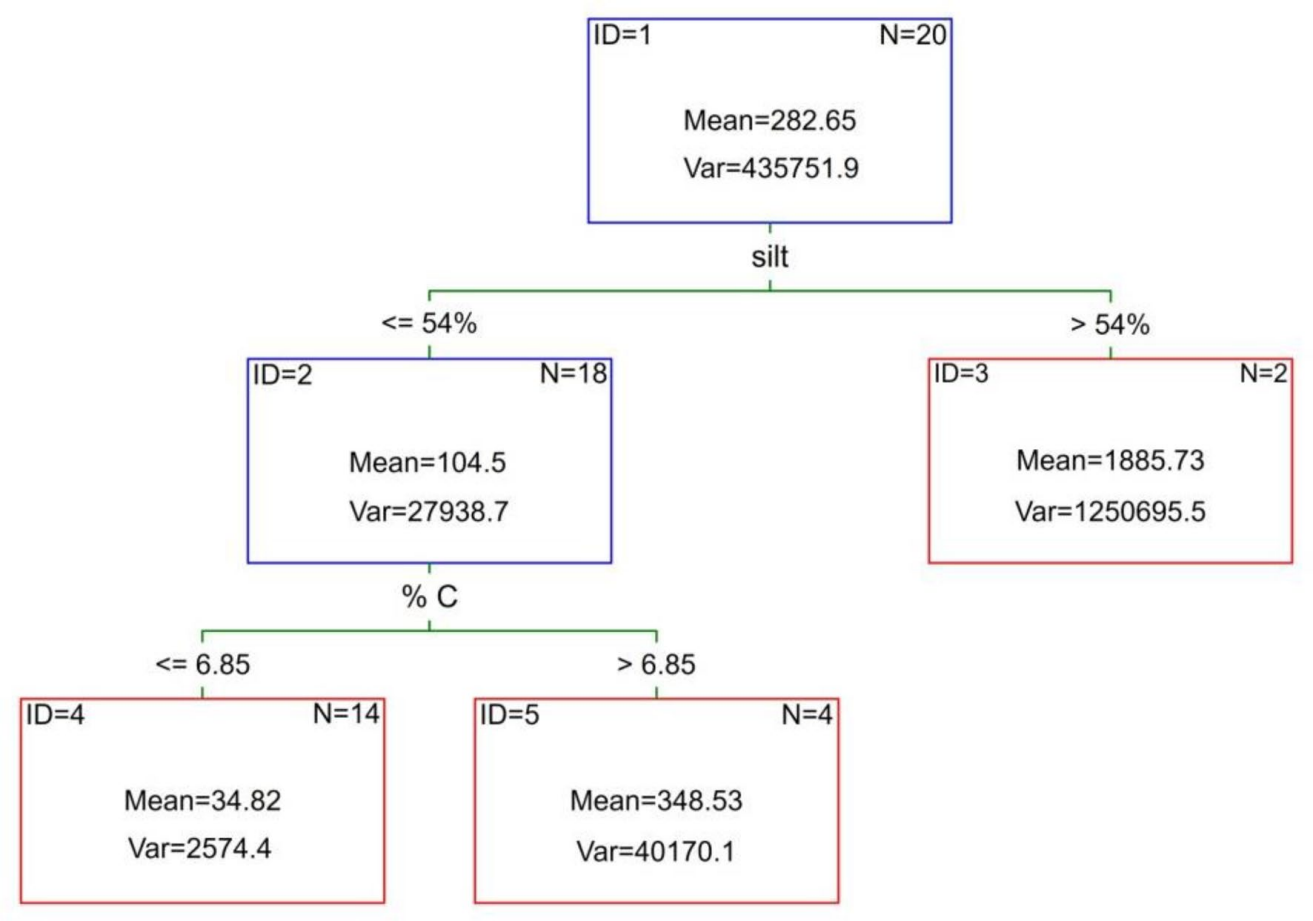
The regression tree (C&RT) for PAHs content in soils, was generated using Statistica 13 software.

**Figure 6:**
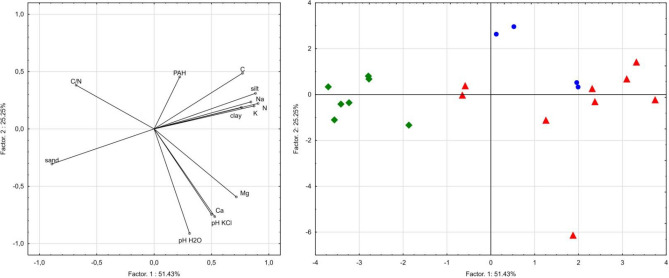
The projection of variables on a plane of the first and second PCA factor (green symbols—sand; red symbols—sandy loam; blue symbols—silt loam), was generated using Statistica 13 software.

The regression tree for PAHs content in soils confirmed the importance of texture in shaping the distribution of PAHs (Fig. 5). The regression tree indicates silt content as a variable explaining the concentration of PAHs in the studied soils. Carbon content in soils determines PAHs distribution in forest soils to a smaller extent (Fig. 5). The PCA analysis confirmed the differences in properties of soils with different texture. Sandy soils form a separate group which is characterized by lower contents of C, N, alkaline cations and PAHs. Soils with heavier texture were characterized by higher PAHs content and more favorable soil properties (Fig. 6). Factors 1 and 2 explain 76.68% of the variability of the examined features. Factor 1 is related to the content of sand, silt, clay and alkaline cations. Factor 2 is related to the content of PAHs in the tested soils (Fig. 6).

## Discussion

The study carried out confirmed the validity of the first hypothesis. Soil texture, especially the silt content, was importance in PAHs distribution in forest soils. A significant correlation of PAHs content in soils with the content of silt fraction was recorded. The share of particular granulometric fractions to a large extent determines the soil sorption properties^20,41^. Previous studies have confirmed the importance of soil texture in PAHs accumulation^42,43^. In soils with heavier texture like sandy loam, silt loam, high content of 4, 5 and 6 ring aromatic hydrocarbons was recorded. The key factors determining the stability of PAHs in soil include their structure and properties^44^. Two and three-ringed hydrocarbons which have a lower molecular weight and higher water solubility are more susceptible to degradation than those with more rings. An increase in the number of rings in PAH molecules increases their molecular weight and hydrophobic properties, thus reducing microbiological degradation^19^. Due to water solubility, 2-ring PAHs and to a lesser extent 3-ring PAHs are more available for biological degradation and uptake^45^. Sorption and degradation are key processes that affect the fate and transport of PAHs in the environment^46^.

When listing the soil properties that determine PAHs accumulation in soils, soil organic matter is indicated first, followed by soil texture. Our study shows that texture is more important in shaping PAHs distribution in forest soil. The regression tree analysis confirmed the importance of silt content, followed by soil organic carbon in PAHs distribution. Many studies demonstrated that PAHs adsorb strongly on the surface of soil particles and organic matter and are therefore not easily biodegradable^17,47^. In our study, we did not note a relationship between soil enzymatic activity and PAHs content in soils. This may be due to the strong binding of PAHs by fine fraction particles, especially silt. Maliszewska-Kordybach and Smreczak noted that high PAHs concentrations did not affect enzymatic activity^48^. This is related to the low solubility of these compounds and consequently low bioavailability to soil biota. Baran et al. noted a stimulating influence of PAHs on dehydrogenase activity and biochemical potential fertility indicator, which was attributed to an adaptation of the soil microflora and the use of pollutant as a C and energy source^49^. When there are significant differences in the amount of soil organic matter, it has a greater effect on enzyme activity than low-level exotic pollutants^50^. The enzymatic activity and microbial biomass of the soils studied correlated very strongly with the carbon and nitrogen content of the soils. There was a strong correlation between the organic carbon content and the enzymes involved in the C and N cycles. Changes in the dynamics of soil microorganisms are related to the availability of substrates^51^. Dou et al. noted a significant relationship between arylsulphatase, phosphatase and β-d-glucosidase activity and C, N and P concentration in soils^52^. Soil organic matter is a carrier of enzymes, affects the microbial biomass and community structure^53,54^. It is revealed that SOC was positively correlated with soil microbial community and organic carbon availability was reported to be major determine for preservation of soil microbial diversity^55^. Nitrogen affects many ecosystem functions, plant community diversity and microbial communities^56^. In our study, we noted the stimulating role of nitrogen on enzymatic activity and microbial biomass C and N. N addition indirectly affects soil enzyme activity through changes in the composition of microbial communities^57^. Summarizing the analyses carried out, the second hypothesis concerning the role of organic carbon in shaping PAHs content and enzymatic activity of forest soils is correct.

The permissible content of heavy metals was exceeded most in soil with sandy loam and silt loam texture. The clay and silt fractions are the main carrier of the soil’s adsorption properties. According to Kabata-Pendias^58^, the admissible level of Cd amounts to 1 mg/kg, Co to 10 mg/kg, Cr to 60 mg/kg, Cu to 30 mg/kg, Ni to 20 mg/kg, Pb to 50 mg/kg and Zn to 100 mg/kg. The metal contents found in uncontaminated soils were not exceeded for manganese, chromium, cobalt and nickel. In our study, we confirmed a strong relationship between heavy metals and soil texture, organic carbon content and the content of alkaline cations. Verla et al. showed that clay particle size had strong influence on heavy metals mobility^59^. Soil organic matter and clay minerals is the main component of soil that possess significant sorption capacity relative to metals through exchange sorption, complexing or chelation^19^. High level of PAHs were recorded in soil with sandy loam and silt loam texture. The sum of PAHs in that soils exceed 100 μg/kg, the threshold values of classification according to Maliszewska-Kordybach criteria^60^. The studied soils with highest PAHs content were dominated by the silt fraction which conducive to PAH accumulation. According to Lu et al. the highest PAH concentration was associated with clay and silt fraction^61^.

Our data set indicate that the mean topsoil value of mass magnetic susceptibility (42 × 10^−8^ m^3^/kg) was higher than the mean topsoil values found by Dearing et al. and Hanesch and Scholger for typical Stagnosol^62,63^. Observed differences might be an effect of particular parent material, soil properties (e.g. soil texture), environmental settings, or impact of industrial emission. Three times higher magnetic susceptibility was determined in topsoil with fine grains (Table 4). This phenomenon was proved by significant (both positive and negative) correlation between magnetic susceptibility and soil texture in topsoil (Table 5), as reported by many other^64–66^. On the other hand, the presence of ultra-fine superparamagnetic grains of pedogenic origin was not confirmed by percentage frequency-dependent magnetic susceptibility measurements^62^. The values of χ_fd_ in the study soil samples were low (i.e. below 2%, Table 4), suggesting the technogenic or geogenic origin of magnetic particles. However, in the case of relatively low values of both χ and χ_fd_, and due to the fact that the study area was located in the industrial emission zone, the presence of geogenic iron minerals ought to be excluded.

## Conclusions

Our analysis confirmed the importance of the soil texture, followed by soil organic carbon in PAHs distribution. High silt content positively affected PAHs content and led to an increase in the PAHs accumulation in forest soil by increasing the sorption capacity of soils. Organic carbon content and nitrogen content stimulates enzyme activity and microbial biomass C and N. Obtained results confirmed that the integrated geochemical and magnetic methods proved to be a useful and effective tool in the assessment of contaminants (in particular heavy metals) of forest soil. Based on this study there is clear evidence that PAH pollution assessments need to consider grain size and soil organic carbon.
